# Bidirectional associations between maladaptive cognitions and emotional symptoms, and their mediating role on the quality of life in adults with ADHD: a mediation model

**DOI:** 10.3389/fpsyt.2023.1200522

**Published:** 2023-07-19

**Authors:** Mei-Rong Pan, Shi-Yu Zhang, Cai-Li Chen, Sun-Wei Qiu, Lu Liu, Hai-Mei Li, Meng-Jie Zhao, Min Dong, Fei-Fei Si, Yu-Feng Wang, Qiu-Jin Qian

**Affiliations:** ^1^Peking University Sixth Hospital, Peking University Institute of Mental Health, Beijing, China; ^2^NHC Key Laboratory of Mental Health (Peking University), National Clinical Research Center for Mental Disorders (Peking University Sixth Hospital), Beijing, China

**Keywords:** attention-deficit/hyperactivity disorder (ADHD), maladaptive cognitions, emotional symptoms, quality of life (QoL), mediation model

## Abstract

**Background/objectives:**

Adults with attention-deficit/hyperactivity disorder (ADHD) have more maladaptive cognitions, emotional problems and a poorer quality of life (QoL). A verification of the psychological model in clinical samples is needed for a better understanding of the mechanisms of ADHD diagnosis on QoL via maladaptive cognitions, emotional symptoms, and their interactions.

**Methods:**

299 ADHD participants and 122 healthy controls were recruited. ADHD core symptoms, maladaptive cognitions, emotional symptoms and psychological QoL were rated. Pearson’s correlation and structural equation modeling were analyzed to explore the relationship and influence of ADHD diagnosis on QoL.

**Results:**

More maladaptive cognitions, emotional symptoms, and poorer QoL were found in the ADHD group, and the dysfunctional attitudes were on par between ADHD with or without medication (*p* = 0.368). Moderate to strong correlations were found between emotional symptoms, maladaptive cognitions and QoL, and ADHD core symptoms presented correlations among the above scores (*r* = 0.157 ~ 0.416, *p* < 0.01) in ADHD participants. The influence of ADHD diagnosis on QoL was mediated through maladaptive cognitions, emotional symptoms, and their bidirectional interactions (*p* < 0.05), especially those with stable medication.

**Conclusion:**

Our study is the first to verify the psychological model in adults with ADHD in China. The findings determined the direct influence of ADHD diagnosis on QoL and the indirect influence through maladaptive cognitions, emotional symptoms, and their interactions, emphasizing the importance of interventions for emotional symptoms and maladaptive cognitions for ADHD patients both with or without medication for a better QoL outcome.

## 1. Introduction

Attention-deficit/hyperactivity disorder (ADHD) is a common, chronic neurodevelopmental disorder defined as a persistent, trans-situational pattern of inattention and/or hyperactivity-impulsivity inappropriate to the developmental stage (1), which affects approximately 4.4–5.2% of adults between 18 and 44 years of age (2, 3). Adults with ADHD may struggle with emotional problems due to the existence of emotional dysregulation (4), risking the occurrence of comorbidities such as bipolar disorder (5), depression (6), anxiety (7, 8), substance abuse (9, 10), addictive behaviors (11, 12), and personality disorders (13, 14). Meanwhile, ADHD patients have a burden on their physical health and academic, social, and occupational functioning (15, 16), and more deficits in quality of life (QoL) over their lifespan (17). Cooccurring emotional symptoms can also affect individuals’ QoL in later life (18).

ADHD patients show elevated dysfunctional cognition scores (19, 20) and more maladaptive schemas (21) than controls. Meanwhile, both behavioral avoidance and dysfunctional cognitions have been found to mediate the relationship between ADHD and a comorbid depression diagnosis (22), and less ruminative thinking patterns and cognitive-behavioral avoidance are protective factors of ADHD-depression comorbidity (23), indicating the mediating role of maladaptive cognitions in the relationship between ADHD and the comorbid emotional disorders.

Maladaptive cognitions also arise when anticipating or experiencing higher levels of unwanted emotions persistently, such as intense worry in anxiety (24), or low mood in depression (25, 26) since the lack of ability to inhibit or down-regulate emotional responses (27), and eventually lead to a vicious cycle. Bidirectional relationships between emotion regulation strategies and mental health symptoms have been found (28), suggesting that maladaptive cognitions may be associated with emotional symptoms (29), and lead to daily life impairment and poor social interactions (30) in ADHD adults. However, their causal interactions with ADHD symptoms and the influences on QoL still need to be explored.

Several researchers explored the psychological model of ADHD (21, 31, 32), indicating that maladaptive cognitions result from early experiences of emotional stress and negative feedback from others (33), emotional neglect or abuse (34) in school, work, and relationships since the existence of ADHD symptoms, such as attentional problems, emotional instability, or impulsivity, which cause and in turn negatively shape the individual’s beliefs, emotions and self-esteem (35). Negative expectations of the future and decreased self-confidence can also affect individuals’ motivation to complete the task, resulting in more failure experiences and frustrations (36) and leading to poor life satisfaction. The theorical model still needs to be verified in clinical samples.

Medication is currently the first-line treatment for adults with ADHD (37), and the efficacy of medication have been proven (38). Whereas, a systematic review figured out that current treatments may not usually ‘normalize’ the ADHD patients. The QoL impairments (39) in medicated ADHD highlights the need for additional interventions to achieve better functional outcomes, such as psychotherapy, which has been found to be effective for quality of life in the follow-ups (40). Thus, a better understanding of the psychological mechanism of ADHD and its influence on QoL in ADHD patients with or without medication in clinical samples may be helpful to provide more empirical evidence for the treatment choice and decision-making in term of psychotherapy for a better functional outcome.

Altogether, adults with ADHD have more maladaptive cognitions and emotional problems, and theorizing suggests they correlate with each other and might lead to poor QoL. Limited studies discussed the bidirectional relationships between maladaptive cognitions and emotional symptoms in adults of ADHD and their influences on QoL. One of the few published studies (41) indicated that more severe ADHD symptoms are associated with higher levels of perceived stress both directly and indirectly through stronger maladaptive cognitions, which, in turn, are related to poor emotional well-being. Torrente et al. (19) found that adults with ADHD scored higher on dysfunctional attitudes than nonclinical participants but were on par with clinical participants, suggesting that dysfunctional cognitions and other diagnoses might be correlated. However, no control group was included in the above studies when exploring factors affecting QoL, so the differences between the ADHD group and the healthy control group could not be examined, and the differences between those with and without medication have not been discussed. Our previous research also found the emotional and QoL impairments in ADHD adults (42), but the role of maladaptive cognitions still need to be explored. Thus, a further study on maladaptive cognitions, emotions, and their influences on QoL would help provide a deeper understanding of the psychological model in adults with ADHD, especially the comparison between the ADHD group and healthy controls and the subgroup differences between those with and those without medication.

In our study, we aimed to explore (1) the relationships among maladaptive cognitions, emotional symptoms and QoL in adults with ADHD, and (2) the possible mechanism of maladaptive cognitions and emotional symptoms between ADHD diagnosis and QoL through a mediation model. We also separately explored the mechanism in samples with and without medication in order to get a better understanding of the psychological model. Based on previous studies and our research experiences, we hypothesized that (1) the correlations among maladaptive cognitions, emotional symptoms, and QoL are significant in adults with ADHD, for both those with and without medication; (2) both maladaptive cognitions and emotional symptoms mediate the relationship between ADHD and QoL; and (3) a bidirectional association exists between maladaptive cognitions and emotional symptoms and mediates the relationship between ADHD and QoL (ADHD → maladaptive cognitions ↔ emotional symptoms → QoL).

## 2. Methods

### 2.1. Sample

The participants were outpatients of Peking University Sixth Hospital and individuals recruited from the internet from March 2019 to September 2022. The key inclusion criteria were as follows:

1. being an outpatient of Peking University Sixth Hospital, aged between 18–45 years, and having received a diagnosis of adult ADHD through Conners’ Adult ADHD Diagnostic Interview (43) based on the Diagnostic and Statistical Manual of Mental Disorders, Fourth Edition (DSM-IV) (44) and a Clinical Global Impression Scale (CGI-S) score ≥ 3.
2. ADHD medication-naïve or have stable use of ADHD medication (drug fluctuations <10% for at least 1 month) (45), either methylphenidate hydrochloride controlled-release tablets (Concerta®) or atomoxetine hydrochloride (Strattera®).

The key exclusion criteria included the following:

1. had a history of schizophrenia or pervasive developmental disorder;
2. had a history of severe external brain injuries or neurological diseases with a loss of consciousness, and other serious somatic diseases;
3. exhibiting high suicide risk;
4. having a full-scale intelligence quotient (FIQ) < 80;

The healthy controls (HCs) were age– and sex-matched with those in the ADHD group. Additionally, they did not meet the criteria for a diagnosis of ADHD based on an evaluation by trained psychiatrists. Those with a history or current diagnosis of neurological or mental illness, family history of mental illness, or serious somatic disease and FIQ < 80 were excluded.

All participants were assessed for mental disorders according to the Structured Clinical Interview for DSM-IV Axis-I (46) and Axis-II (47). The FIQ was obtained from the Wechsler Adult Intelligence Scale-Revised in China, Third Edition (WAIS-RC) (48). Eventually, 299 adult ADHD patients and 122 HCs were included. All participants were informed of the purpose of the study and were told that the study data would be aggregated. The study was approved by the Ethics and Clinical Research Committees of Peking University Sixth Hospital, and all participants signed an informed consent form.

### 2.2. Measures

The ADHD Rating Scale (ADHD-RS) (49) was used to assess the ADHD core symptoms. The Self-rating Depression Scale (SDS) (50) and Self-rating Anxiety Scale (SAS) (51) were used to estimate current emotional symptoms. The Automatic Thoughts Questionnaire (ATQ) (52) and Dysfunctional Attitude Scale (DAS) (53) were used to estimate individuals’ maladaptive cognitions. Additionally, we used the Brief Version of the World Health Organization Quality of Life Scale (WHOQOL-BREF)-psychological domain score to estimate the psychological quality of life (QoL- psychological domain) (54).

All participants underwent diagnostic interviews and FIQ evaluations by assessors who had received unified training on all the measurement tools, and the consistency was rated. The ethics committee protected the interests of the subjects, such as safety and confidentiality.

### 2.3. Statistical analysis

Independent two-sample t tests and chi-square (χ2) tests were used to compare the baseline variables between the ADHD and HC groups. We divided the ADHD participants into a group with ADHD medication (the medicated ADHD group) and a group without medication (the non-medicated ADHD group) according to the stable use of ADHD medication. One-factor analysis of variance (one-way ANOVA) and χ2 tests were used to compare the differences among the medicated, non-medicated and HC groups. The Bonferroni method or χ2 tests were used for the *post hoc* test. Pearson’s correlation was used to assess the correlation among clinical variables, including ADHD core symptoms (ADHD-RS), emotional symptoms (SAS and SDS), maladaptive cognitions (ATQ and DAS) and QoL (WHOQOL-BREF psychological domain score), in the whole, medicated and non-medicated ADHD groups. Irrelevant, weak, moderate, and strong correlations (*r*) were defined as *r* values of 0 ~ 0.09, 0.10 ~ 0.30, 0.30 ~ 0.50, and 0.50 ~ 1.00, respectively.

**Figure 1:**
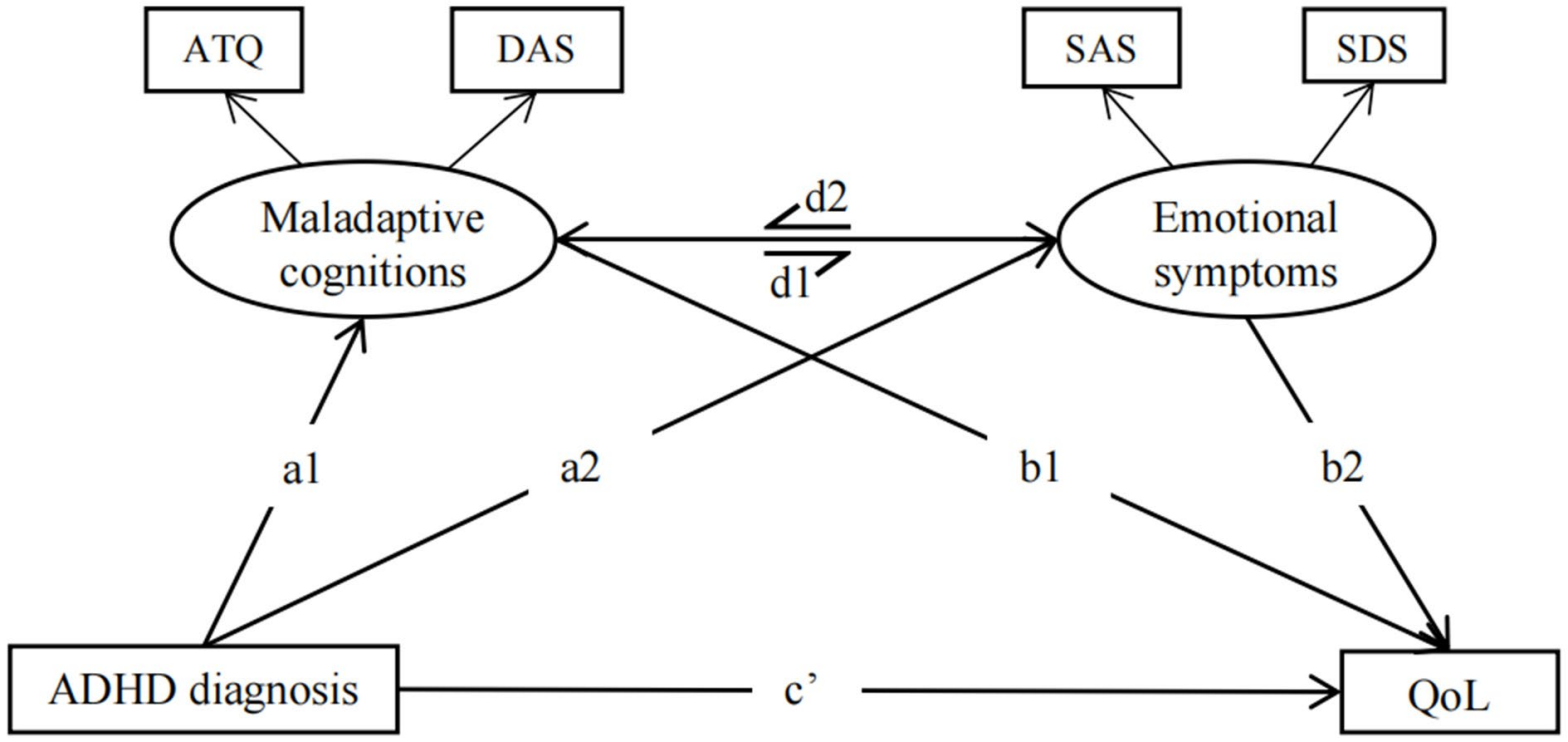
Hypothetical model to test the direct and indirect effects of ADHD diagnosis on QoL *via* maladaptive cognitions and emotional symptoms. Maladaptive cognitions included ATQ and DAS total scores; Emotional symptoms included SAS and SDS; ATQ: Automatic Thoughts Questionnaire; DAS, Dysfunctional Attitudes Scales; SAS, Self-Rating Anxiety Scale; SDS, Self-Rating Depression Scale; Qol, Psychological quality of life: World Health Organization Quality of Life Scale (WHOQOL-BREF)-psychological domain score.

Structural equation mediation model analyses (SEM) were performed using the R package lavaan (55) with the R software (Version 4.2.2) to test the direct and indirect effects of ADHD diagnosis on QoL via maladaptive cognitions and emotional symptoms. Based on the bidirectional relationships between maladaptive cognitions and emotional symptoms, a mediation analysis model “ADHD diagnosis→maladaptive cognitions ↔ emotional symptoms→QoL” was constructed as shown in Figure 1. Structural models were used separately in the medicated ADHD group and the non-medicated ADHD group to figure out the differences between the groups. All mediation analyses were controlled for baseline dimension indicators (such as age, gender, years of education, FIQ, etc.) if differences between groups were found. Model fit was assessed using the confirmatory fit index (CFI) (56), root mean square error of approximation (RMSEA), and standardized root mean square residual (SRMR) (57).

## 3. Results

### 3.1. Clinical characteristics of the ADHD group

A total of 299 ADHD participants were recruited, with 170 (56.86%) diagnosed with the predominantly inattentive subtype (ADHD-I), and the others diagnosed with the combined subtype (ADHD-C). 177 (59.20%) participants were diagnosed with comorbidities, including bipolar disorder (BD) (34, 11.37%), affective disorders (116, 38.80%), anxiety disorders (88, 26.42%), eating disorders (11, 3.68%), and substance use disorders (SUD) (6, 2.01%). All ADHD participants with comorbidities, especially those with SUD or BD, persisted in complete remission, and the use of ADHD medications was prescribed based on the clinical demand to confirm the efficacy and safety of medication treatment. 189 patients reported stable use of ADHD medication, including methylphenidate (159, 53.18%), atomoxetine (29, 9.70%), or both (1, 0.33%). Among the 189 medicated patients, 34 (11.37%) were co-administered with other psychiatric medications, including antidepressants, mood stabilizers, and antipsychotics.

### 3.2. The differences between the ADHD group and the HC group in clinical characteristics

No significant differences were found between the ADHD group and the HC group in sex and age. The FIQ and years of education in the ADHD group were significantly lower than that in the HC group (*p* = 0.000). The ADHD-RS total, SAS and SDS scores were significantly higher in the ADHD group than in the HC group (*p* = 0.000). The ATQ and DAS total scores in the ADHD group were significantly higher than those of the HC group, and the WHOQOL-psychological domain score was significantly lower in the ADHD group than in the HC group (*p* < 0.001) (Table 1).

**Table 1:** Demographic information and clinical characteristics between the ADHD group and the HC group.

	ADHD group (*n* = 299)	HC group (*n* = 122)	*χ^2^/t value*	*p* value
Male (%)	152 (50.84%)	54 (44.26%)	1.498	0.221
Age	26.51 ± 5.62	25.66 ± 3.24	1.947	0.052
FIQ	120.6 ± 9.42	124.3 ± 6.97	−4.421	0.000***
Years of education	16.25 ± 2.57	18.06 ± 1.98	−6.952	0.000***
ADHD-RS	27.31 ± 9.51	5.93 ± 4.53	31.153	0.000***
SAS	43.63 ± 10.74	31.61 ± 5.71	14.863	0.000***
SDS	48.97 ± 12.75	33.16 ± 6.62	16.646	0.000***
ATQ total score	69.9 ± 23.84	37.19 ± 8.38	20.763	0.000***
DAS total score Total score	147.84 ± 34.89	108.44 ± 24.65	13.086	0.000***
WHOQOL-BREF Psychological domain	44.93 ± 17.25	75.58 ± 13.23	−19.660	0.000***

HC group, the healthy control group; FIQ, full-scaled intelligence quotient; ADHD-RS, ADHD-Rating Scale; SAS, Self-Rating Anxiety Scale; SDS, Self-Rating Depression Scale; WHOQOL-BREF, World Health Organization Quality of Life-Brief Version; ***: *p* < 0.001.

When comparing the differences among the medicated, non-medicated, and HC groups, we found that there were significant differences in all scores above among the three groups. *Post hoc* pairwise comparisons within each group indicated that the non-medicated ADHD group had significantly higher SAS, SDS, ATQ scores, and significantly lower WHOQOL-psychological domain score than the medicated ADHD group. No difference was found in DAS total score between the medicated and the non-medicated ADHD groups (*p* = 0.368) (Table 2).

**Table 2:** The differences among the non-medicated ADHD group, medicated ADHD group, and the HC group in clinical characteristics.

	Non-medicated ADHD group (*N* = 110)	Medicated ADHD group (*N* = 189)	HC group (*N* =122)	*F value*	*post hoc* *t* test
ADHD-RS	28.84 ± 7.72	26.42 ± 10.34	5.92 ± 4.55	254.710***	Non-medicated ADHD* > Medicated ADHD*** > HC
SAS	48.43 ± 10.19	40.93 ± 10.08	31.65 ± 5.72	87.724***	Non-medicated ADHD*** > Medicated ADHD*** > HC
SDS	54.44 ± 12.63	45.80 ± 11.77	33.13 ± 6.64	99.293***	Non-medicated ADHD*** > Medicated ADHD*** > HC
ATQ total score	74.18 ± 24.63	67.43 ± 23.08	37.07 ± 8.30	100.258***	Non-medicated ADHD* > Medicated ADHD*** > HC
DAS total score	151.83 ± 36.49	145.54 ± 33.82	108.21 ± 24.61	54.740***	Non-medicated ADHD > Medicated ADHD*** > HC
WHOQOL-BREF Psychological domain	41.09 ± 16.05	47.00 ± 17.53	75.69 ± 13.24	137.325***	Non-medicated ADHD** < Medicated ADHD*** < HC

HC group: the healthy control group; SAS, Self-Rating Anxiety Scale; SDS, Self-Rating Depression Scale; ATQ, Automatic Thoughts Questionnaire; DAS, Dysfunctional Attitudes Scales; WHOQOL-BREF, World Health Organization Quality of Life-Brief Version; *: *p* < 0.05; **: *p* < 0.01; ***: *p* < 0.001.

### 3.3. Relationships between ADHD core symptoms, emotional symptoms, maladaptive cognitions, and QoL in adults with ADHD

Correlation analyses were used to evaluate the relationships in adults with ADHD after controlling for sex, age, FIQ and years of education. Positive correlations between ADHD-RS and SAS, ATQ, DAS (*r* = 0.157 ~ 0.416, *p* < 0.001) and a negative correlation with WHOQOL-BREF-psychological domain (*r* = −0.209, *p* < 0.001) were found, and the correlations were small to moderate. Emotional symptoms (SAS and SDS) were positively correlated with maladaptive cognitions (ATQ and DAS) (*r* = 0.381 ~ 0.618, *p* < 0.001) and negatively correlated with the WHOQOL-BREF-psychological domain (*r* = −0.480 and − 0.643, *p* < 0.001, respectively), and the correlations were moderate to strong. When controlling for the use of medication, the correlation still existed (Table 3).

**Table 3:** The correlations among the ADHD core symptoms, emotional symptoms, maladaptive cognitions, and QoL in adults with ADHD.

	Without controlling for medication					With controlling for medication				
	ADHD-RS	ATQ	DAS	SAS	SDS	ADHD-RS	ATQ	DAS	SAS	SDS
ATQ	0.275***					0.263***				
DAS	0.157**	0.572***				0.147*	0.568***			
SAS	0.416***	0.522***	0.381***			0.397***	0.515***	0.376***		
SDS	0.264***	0.618***	0.396***	0.811***		0.235***	0.616***	0.391***	0.788***	
WHOQOL-BREF Psychological domain	−0.209***	−0.695***	−0.415***	−0.480***	−0.643***	−0.191***	−0.690***	−0.408***	−0.459***	−0.634***

QoL, quality of life; ADHD-RS, ADHD-Rating Scale; SAS, Self-Rating Anxiety Scale; SDS, Self-Rating Depression Scale; ATQ, Automatic Thoughts Questionnaire; DAS, Dysfunctional Attitudes Scales; WHOQOL-BREF, World Health Organization Quality of Life-Brief Version; *: *p* < 0.05; **: *p* < 0.01; ***: *p* < 0.001.

### 3.4. Mediation analyses

#### 3.4.1. Structural equation mediation models in the whole ADHD group

The structural model for ADHD diagnosis on QoL in the whole ADHD group compared with the HC group showed good fit (χ2 (df = 8.000) =15.076, *p* = 0.058, CFI = 0.996, RMSEA = 0.046, SRMR = 0.013). The mediation effect model showed that the direct (c’ = −8.164, *p* = 0.000) and total effect (c = −58.068, *p* = 0.000) of ADHD diagnosis on the WHOQOL-psychological domain score were significant. The indirect effect through maladaptive cognitions (a1b1 = −3.032, *p* = 0.012) and emotional symptoms (a2b2 = −13.956, *p* = 0.002) were also significant. The indirect effect of ADHD diagnosis on QoL was statistically significant both through maladaptive cognitions and then emotional symptoms (a1d1b2 = 1.640, *p* = 0.035), and also through emotional symptoms and then maladaptive cognitions (a2d2b1 = −34.556, *p* = 0.000). When controlling for the use of medication, all the mediation paths existed (*p* = 0.000) except the mediation role through maladaptive cognitions and then emotional symptoms did not exist (a1d1b2 = 0.459, *p* = 0.123).

#### 3.4.2. Structural equation mediation models in the medicated and non-medicated ADHD group

**Figure 2:**
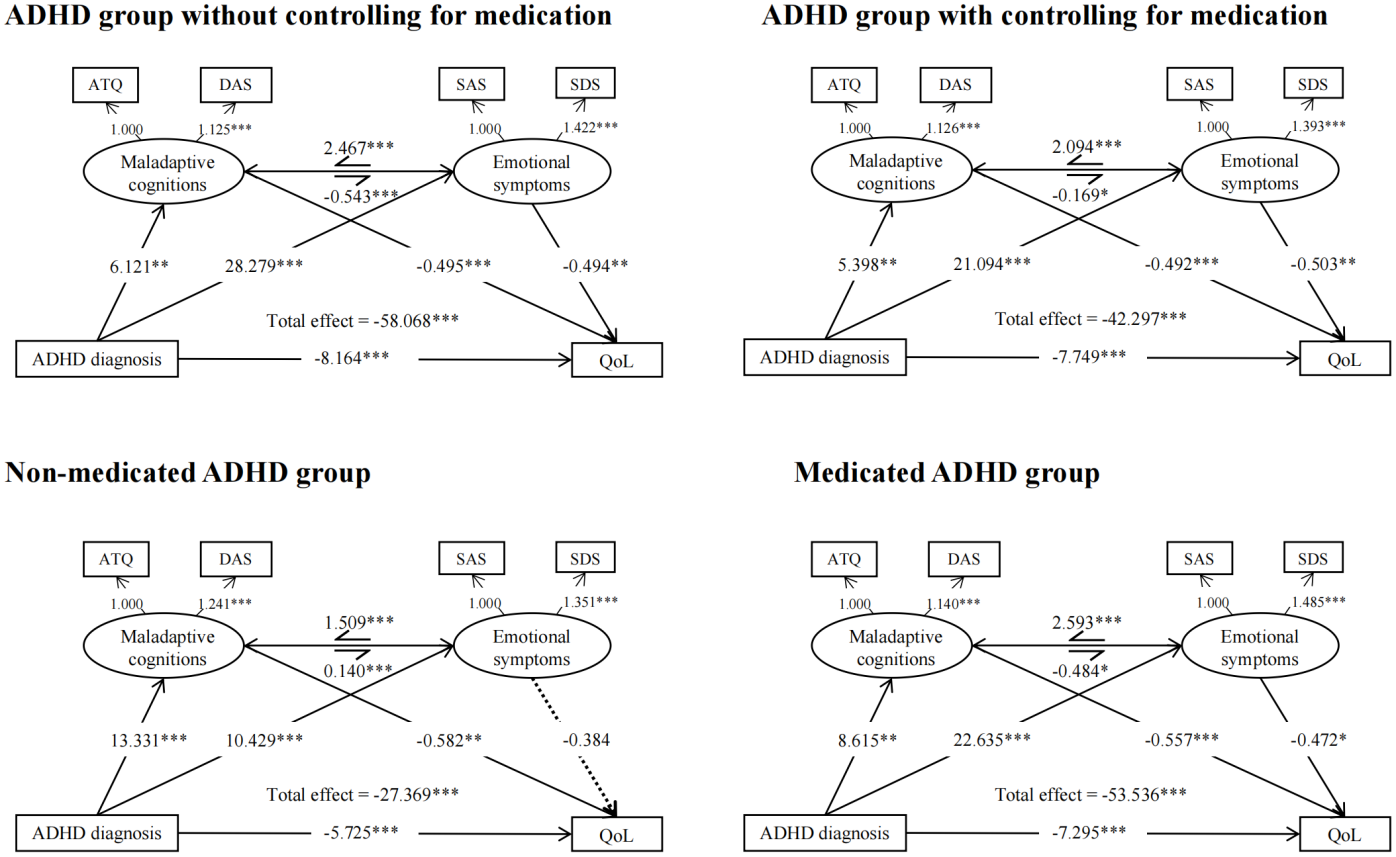
Structural equation mediation models of ADHD diagnosis on QoL *via* maladaptive cognitions and emotional symptoms. SAS, Self-Rating Anxiety Scale; SDS, Self-Rating Depression Scale, ATQ, Automatic Thoughts Questionnaire, DAS, Dysfunctional Attitudes Scales, Qol, Psychological quality of life: World Health Organization Quality of Life Scale (WHOQOL-BREF)-psychological domain score. ***p < 0.001, **p < 0.01, *p < 0.05.

We then examined the mediation effect separately in the medicated and non-medicated ADHD groups compared with the HC group, and found good fit in both models (the mediated ADHD group: χ2 (df = 10.000) = 13.945, *p* = 0.176, CFI = 0.997, RMSEA =0.036, SRMR = 0.014, the non-medicated ADHD group: χ2 (df = 8.000) = 12.731, *p* = 0.121, CFI = 0.997, RMSEA = 0.051, SRMR = 0.011). The significant direct effect, total effect, and indirect effect through maladaptive cognitions, or through emotional symptoms and then maladaptive cognitions were found in both groups (*p* < 0.05). The indirect effect through emotional symptoms were significant only in the medicated ADHD group (a2b2 = −10.683, *p* = 0.039) but not in non-medicated ADHD group (*p* = 0.369). A trend of significant indirect effect through maladaptive cognitions and then emotional symptoms could be found in the medicated ADHD group (a1d1b2 = 1.966, *p* = 0.065). The indirect effect of ADHD through emotional symptoms did not exist dependently (a2b2 = −4.002, *p* = 0.369), but existed through emotional symptoms and then the maladaptive cognitions (a2d2b1 = −9.162, *p* = 0.006) in the non-medicated ADHD group (Table 4 and Figure 2).

**Table 4:** Structural equation mediation models of ADHD diagnosis on QoL *via* maladaptive cognitions and emotional symptoms.

		Estimate	Standard error	*p* value	Ratio of mediating effect
ADHD group without controlling for medication					
c’		−8.164	1.905	0.000***	14.06%
a1b1		−3.032	1.211	0.012*	5.69%
a2b2		−13.956	4.464	0.002**	24.03%
a1d1b2		1.640	0.779	0.035*	2.82%
a2d2b1		−34.556	7.961	0.000***	59.51%
c		−58.068	6.566	0.000***	
ADHD group with controlling for medication					
c’		−7.749	2.055	0.000***	18.32%
a1b1		−2.657	0.737	0.000***	6.28%
a2b2		−10.612	3.447	0.002**	25.09%
a1d1b2		0.459	0.297	0.123	1.09%
a2d2b1		−21.738	5.762	0.000***	51.39%
c		−42.297	5.368	0.000***	
Non-medicated ADHD group					
c’		−5.725	2.779	0.039*	20.92%
a1b1		−7.762	2.841	0.006**	28.36%
a2b2		−4.002	4.452	0.369	14.62%
a1d1b2		−0.718	0.766	0.348	2.62%
a2d2b1		−9.162	3.340	0.006**	33.48%
c		−27.369	2.503	0.000***	
Medicated ADHD group					
c’		−7.295	2.567	0.004**	13.63%
a1b1		−4.803	2.193	0.029*	8.97%
a2b2		−10.683	5.188	0.039*	19.95%
a1d1b2		1.966	1.067	0.065.	3.67%
a2d2b1		−32.721	13.576	0.016*	61.12%
c		−53.536	13.567	0.000***	

c’: Direct effect; a1b1: ADHD → Maladaptive cognitions → QoL; a2b2: ADHD → Emotional symptoms → QoL; a1d1b2: ADHD→ Maladaptive cognitions →Emotional symptoms → QoL; a2d2b1: ADHD→ Emotional symptoms → Maladaptive cognitions → QoL; c: Total effect; *: *p* < 0.05; **: *p* < 0.01; ***: *p* < 0.001.

## 4. Discussion

Our research yielded the following findings. First, more emotional symptoms, maladaptive cognitions, and poorer QoL were found in the ADHD group, both with and without medication. Second, ADHD patients without medication presented more ADHD core symptoms, emotional symptoms, automatic thinking and poor psychological QoL compared with those with medication, and the dysfunctional attitudes remained no differences. In addition, the ADHD core symptoms, emotional symptoms, maladaptive cognitions and psychological QoL were correlated whether controlling for the use of medication or not. Then, the influence of ADHD diagnosis on QoL was mediated through maladaptive cognitions and emotional symptoms, and the bidirectional associations between maladaptive cognitions and emotional symptoms. The use of ADHD medication may reduce the influence on QoL via maladaptive cognitions associated with ADHD since we found the mediation role through maladaptive cognitions and then emotional symptoms was not significant when controlling for the use of medication. A trend of influence of ADHD diagnosis on QoL through maladaptive cognitions and then emotional symptoms still existed in medicated ADHD group, again verified that the use of medication does not fully decrease the dysfunctional attitudes, and the existence of maladaptive cognitions may still increase the risk of emotional distress and then QoL impairments.

This study was the first to explore the maladaptive cognitions of adult ADHD in the Chinese population, and the results are consistent with previous findings (19, 58). The most common cognitive distortions encountered in the adults with ADHD include perfectionism (59), all-or-nothing thinking, magnification or minimization, and comparative thinking (31). The emotional symptoms and QoL impairment of ADHD have also been found in previous studies (60, 61).

Compared with those without medication, ADHD patients with stable medication were found to have less ADHD core symptoms, emotional symptoms, and more life satisfaction. Efficacy of medication has been proven in ADHD core symptoms (62), the comorbid emotional symptoms associated with ADHD (63), and life quality (64). Whereas, patients with stable medication presented significant improvement only in negative automatic thinking but not in dysfunctional attitudes in our study, indicating the limitation of phamarchotherapy in maladaptive cognitions. Compared with the healthy controls, the impairment of quality of life still existed when patients achieved stable medication, consisted with previous findings (65), emphasizing the importance of further intervention of ADHD, especially in the areas of maladaptive cognitions and functional outcomes.

In our study, the ADHD core symptoms, maladaptive cognitions, and emotional symptoms are all correlated with impairments of psychological life quality. The correlation between emotional symptoms and QoL in the ADHD group was moderate to strong, consistent with the findings of bidirectional associations of emotional symptoms and QoL (66–69). Meanwhile, a direct relationship between dysfunctional attitudes and QoL has been established in both clinical and nonclinical population samples (70), with a moderate to strong correlation in our study, indicating the importance of subjective attitude toward individual’s life functioning and satisfaction. Besides, ADHD core symptoms severity was found to have weak to moderate correlation with emotional symptoms, maladaptive cognitions, and QoL, consistent with the previous studies of Stickley et al. (71, 72). Studies figured out the correlation between ADHD core symptoms severity and maladaptive cognitions, since the severity of ADHD symptoms was associated with an increase in internalizing and externalizing problems as well as an increase in perfectionism (73), and personality traits such as perfectionism, dependency and anxiety were significantly associated with ADHD (74). The relative strong correlations among maladaptive cognitions, emotional symptoms and QoL suggested that the existence of emotional symptoms may independently influence maladaptive cognitions and QoL.

We first explored the possible mechanism connecting adult ADHD diagnosis and QoL through the mediators of maladaptive cognitions and emotional symptoms. The mediation analysis found the direct and indirect influences of ADHD diagnosis on QoL via bidirectional associations between maladaptive cognitions and emotional symptoms, consistent with the theoretical psychological model in adult ADHD (21, 31, 32), emphasizing the two identified pathways linking ADHD diagnosis and psychological QoL: the existence of ADHD leads to poor well-being via more emotional stress and maladaptive cognitions, and the pathway of interaction of higher perceived stress and maladaptive attitudes.

Studies found that unwanted intrusive and worrisome thoughts may trouble patients’ anxiety symptoms (75), and negative thoughts (22) as well as cognitive emotion regulation (76) was associated with depressive symptomatology. Additionally, recent findings from cross-sectional studies suggest that poor emotion regulation (77) may mediate the relationship between ADHD and depressive symptoms. The above studies indicated that maladaptive cognitions play an important role in the increased risk of emotional disorders in ADHD patients.

Accumulated studies supported the proposed association between adult ADHD and maladaptive cognitions of failure, combatting stigma, being different from others, and their influences on self-efficacy and self-esteem (31). Researches perceived the development of a negative self-belief as the core issue for maladaptive schema or “secondary symptoms” of stress, emotions, and chronic perceived failure attributed to a history of unachieved potential and negative feedback resulting from a lack of recognition of ADHD (78). Due to the core symptom impairments, patients with ADHD often receive negative feedback from others, which results in accumulation of negative emotions and negative self-concept. This situation causes individuals with ADHD to develop negative coping strategies against the environment and to give negative feedback to their environment. Moreover, ongoing negative feedbacks can also reinforce negative emotions and dysfunctional beliefs, which consequently lead to a vicious cycle (21, 31, 32). As a result, ADHD patients often endure stigmatization (79, 80), violence exposure (81), mental health discrimination (82), and face problems in their education, work, family and social lives (83), which might lead to poor life satisfaction and emotional problems (84, 85). A study in college ADHD students found that negative self-concept and depression fully mediated the association between past academic functioning and self-reported overall functioning at follow-up (86), further verifying the important precursor role of negative self-concept in emotional symptoms in adults with ADHD, which itself originated from the experience of living with ADHD.

When controlling for the use of medication, we found the mediation role through maladaptive cognitions and then emotional symptoms was not significant, indicating that the use of medication reduces the influence on QoL via maladaptive cognitions associated with ADHD. Whereas, the existence of emotional symptoms still influences QoL through maladaptive cognitions, since patients with stable medication still had more emotional symptoms compared with the healthy controls. We then compared the medicated and non-medicated ADHD groups, and found the different mediation roles of maladaptive cognitions and emotional symptoms in the two subgroups. Compared with ADHD patients with medication, those without medication got a higher mediation ratio on QoL via maladaptive emotions related to ADHD. ADHD with stable medication got higher mediation ratio on QoL through emotional symptoms as well as through emotional symptoms and then maladaptive cognitions, suggesting that the existence of emotional symptoms in patients after stable medication will affect quality of life directly or indirectly through maladaptive cognitions. Besides, a trend of mediation effect through maladaptive cognitions and then emotional symptoms can also be found in those with stable medication, since the use of medication does not fully decrease maladaptive cognitions, especially the dysfunctional attitudes directly, and the existence of maladaptive cognitions may still increase the risk of emotional distress. Thus, a further work in individuals’ maladaptive cognitions and emotional distress is important for a better functional outcome in adults with ADHD.

Our study further confirmed the psychological model of ADHD in clinical samples, and emphasized the importance of emotional symptoms and maladaptive cognitions on the influence of QoL. Similar results could be found in other clinical researches (19), suggesting the important part for a better QoL outcome via reduction of ADHD core symptoms, emotional symptoms and related maladaptive cognitions. Combined with the findings regarding the mediation analysis between the medicated and non-medicated ADHD subgroups, we further verified the bidirectional associations between maladaptive cognitions and emotional symptoms, and their influences on QoL in ADHD, emphasizing the importance of influence on QoL outcome via residual emotional symptoms and maladaptive cognitions in ADHD patients with stable medication. The use of medication reduces individual’s core symptoms and the related emotional distress, whereas the impairment of QoL and dysfunctional attitudes still exist, and the residual emotional symptoms and maladaptive cognitions are still target intervention directions in avoidance of the existence of a vicious cycle and their QoL impairment.

CBT has been found to be effective for emotional symptoms, maladaptive cognitions and QoL based on our researches (87–89). Previous studies also emphasized the role of cognitive emotion regulation strategies contributing to resilience of emotional symptoms (90), and the mediation role of dysfunctional attitude reduction in CBT for depressive (91) and anxiety symptoms (92, 93), and success in increasing QoL through CBT (94) has been found. Combined with the verification of the psychological model in ADHD patients obtained in this study, we may have a further understanding of CBT treatment for adult ADHD and its important role on the QoL improvement. The reframing of adaptive cognitions to reduce the distress of emotional symptoms are important.

Our study also had some limitations. The evaluation of patients’ maladaptive cognitions focuses on negative automatic thinking (ATQ) and dysfunctional beliefs (DAS), which are common scales for evaluating patients’ negative thinking and dysfunctional attitudes and have been applied many times in ADHD patients (19, 23) but are not specific for ADHD. ADHD-specific cognitive patterns can be further studied and explored in follow-up studies since researchers have noticed the cognitive pattern of maladaptive positive cognitions in adult ADHD (95) and the ability to positively reappraise stressful situations (96). Besides, our study only explored the relationships and the possible mechanism of ADHD diagnosis on QoL from a cross-sectional perspective. Longitudinal studies are necessary for a further understanding of the causal relationships of the ADHD core symptoms, emotional distress, maladaptive cognitions, and QoL outcomes in the ADHD groups. In addition, the coping strategies and compensatory behavior in ADHD should be further explored in ADHD groups for a better understanding of resilience against ADHD. Last but not least, the participants were mostly outpatients in clinics and individuals recruited from the internet, who would have more needs to be diagnosed and treated, and the sample may not present the whole adult ADHD population in China. Future studies would include more ADHD participants from multi-center clinics and a broader range of education levels for a deeper exploration.

## 5. Conclusion

This study was the first to investigate the maladaptive cognitions of adults with ADHD in China, and found defects in maladaptive cognitions, emotional symptoms, and reduced psychological QoL both with or without stable medication. Our study further validated the psychological model of ADHD in a Chinese population with the use of clinical samples and determined the direct influence of ADHD diagnosis on QoL and the indirect influence through maladaptive cognitions, emotional symptoms, and their bidirectional interactions. Results further emphasized the importance of interventions for emotional symptoms and maladaptive cognitions in patients with ADHD both with or without medication for a better QoL outcome, and provided a theoretical basis for the subsequent development of precision treatment strategies based on the individual and psychological characteristics of patients with ADHD.

## Data availability statement

The original contributions presented in the study are included in the article/supplementary material, further inquiries can be directed to the corresponding author.

## Ethics statement

The studies involving human participants were reviewed and approved by This trial has been approved by the Ethics and Clinical Research Committees of Peking University Sixth Hospital [(2018) Ethics review number (41)] and will be performed in accordance with the Declaration of Helsinki with the Medical Research Involving Human Subjects Act (WMO). The patients/participants provided their written informed consent to participate in this study.

## Author contributions

M-RP and Q-JQ: conceptualization. M-RP, S-YZ, and Q-JQ: design and methodology. M-RP, S-YZ, C-LC, S-WQ: conduction of the study. M-RP, S-WQ, M-JZ, MD, and F-FS: statistical analysis and interpretation. M-RP: writing—original draft preparation. LL, H-ML, Y-FW, and Q-JQ: writing—review and editing. Q-JQ: resources. Y-FW and Q-JQ: supervision. All authors contributed to the article and approved the submitted version.

## Funding

This work was supported by the Capital’s funds for Health Improvement and Research (CFH: 2020–2-4112) and the Beijing Nova Program (20220484061).

## Conflict of interest

The authors declare that the research was conducted in the absence of any commercial or financial relationships that could be construed as a potential conflict of interest.

## Publisher’s note

All claims expressed in this article are solely those of the authors and do not necessarily represent those of their affiliated organizations, or those of the publisher, the editors and the reviewers. Any product that may be evaluated in this article, or claim that may be made by its manufacturer, is not guaranteed or endorsed by the publisher.
